# Sugar Alcohol-Based Deep Eutectic Solvents as Potato Starch Plasticizers

**DOI:** 10.3390/polym11091385

**Published:** 2019-08-23

**Authors:** Magdalena Zdanowicz, Piotr Staciwa, Roman Jędrzejewski, Tadeusz Spychaj

**Affiliations:** 1 Polymer Institute, Faculty of Chemical Technology and Engineering, West Pomeranian University of Technology Szczecin, Ul. Pulaskiego 10, 70-322 Szczecin, Poland; 2 Institute of Materials Engineering, Faculty of Mechanical Engineering and Mechatronics, West Pomeranian University of Technology Szczecin, Al. Piastow 10, 70-310 Szczecin, Poland

**Keywords:** deep eutectic solvents, extrusion, plasticization, polyols, starch, sugar alcohols, thermocompressing molding

## Abstract

The aim of this work was to prepare sugar alcohol-based deep eutectic solvents (DES) and test them as starch plasticizers. Thermoplastic starch (TPS) films were obtained via a simple and convenient thermocompression method. Influence of starch/DES premixtures conditioning (preheating, storage time) on TPS properties was investigated. TPS/sorbitol (S)-based DES exhibited similar tensile strength (TS) (8.6 MPa) but twice higher elongation at the break (*ε*) (33%) when compared with TPS plasticized only with S. Extra treatment, i.e., heating or prolonged storage time, facilitated starch/DES plasticizing. Starch with selected DES was also extruded and the influence of preconditioning and extrusion rotational speed were subsequently studied on thermocompressed films. Extrusion at 100 rpm led to films with TS up to ca. 10 MPa and *ε* up to 52%. Some differences in film samples morphology obtained via two processing methods were observed. X-ray diffractograms revealed that extruded samples exhibited a V-type peak at 18.2°, with intensity depending on plasticizer total molecular size. Applied techniques (mechanical tests, XRD, Dynamic Mechanical Analysis (DMA), FTIR-Attenuated Total Reflection (ATR), and moisture sorption) indicated that S-based DES forms stronger interactions with starch than glycerol (G) only used as conventional plasticizer, thus leading to better mechanical properties and inhibited tendency to starch recrystallization (studied up to one year).

## 1. Introduction

Starch is one of the most abundant natural polymers containing two fractions of α-d-glucose unit: amylose and amylopectin. Amylose is almost completely linear chains mainly based on α(1–4) bonds with molar mass of 10^5^–10^6^ g/mol. Amylopectin is highly branched polymer linked with α(1–4) and α(1–6) bonds with molar mass 10^7^–10^9^ g/mol. Amylose/amylopectin content in starch can be varied from <1% to a little above 80%, depending on the starch source [1]. On the one hand, reactivity of their hydroxyls leads to wide spectra of production possibilities of derivatives with different applications [2,3], but on the other, strong interaction between chains and glass transition temperature (*T*_g_) of unmodified starch near its degradation hinder its processing as conventional thermoplastic materials. For that reason, it requires plasticizer to lower the *T*_g_. Starch plasticizers are polar media that disrupt external OH hydrogen bondings between chains forming themselves new bonds with the polysaccharide [4]. The most common plasticizers are water and glycerol (G), but compounds, such as other polyols or their derivatives, sugars [5,6,7,8,9,10,11,12] amides, and amines [13], or ionic liquids (IL) [14,15], are also applied. However, the types of plasticizers mentioned above exhibit some drawbacks: G allows to polysaccharide recrystallization and, consequently, leads to stiffness of the material; in turn, amines, amides, and IL can be toxic, additionally, the latter are still expensive. A new class of media: deep eutectic solvents (DES) are considered as alternative for IL [16]. They are mixtures with transition temperature (melting or glass transition) much lower than their individual components [16,17]. DES are easy to prepare, cheap, tailorable, and can be made with natural non-toxic compounds. So far, some reports about DES as starch plasticizers have appeared [15,18,19,20,21,22,23,24,25].

Starch plasticization can be performed via: (i) casting from aqueous system after starch gelatinization (typically with G used as the second plasticizer) or melt processable techniques, i.e., (ii) thermocompression (with high temperature and pressure) directly from starch/plasticizer premixture, (iii) extrusion, i.e., mixing and shear forces action at high temperature. Afterwards, extrudate can be processed via thermocompression, melt blowing, or injection molding. The last methods are especially convenient to processing a large amount of material, e.g., in industrial scale (with no requirement of solvent application).

Sugar alcohols, especially sorbitol (S), were used as starch plasticization, but mainly in the casting method [9,10,11,12]. S alone was not an efficient plasticizer for wheat starch processed via thermocompression [13] nor required extra pretreatment at high temperature (150 °C) and mixing for corn starch before thermocompression molding [5]; however, partial gelatinization of the starch system after heating can hinder proportion into extruder. The aim of this work was to prepare and characterize different sugar polyol-based DES and test their plasticizing ability for potato starch (PS), as well as to compare relevant thermoplastic starches (TPS) to materials plasticized with polyol alone. For DES preparation sugar based polyols: S, xylitol (X), and maltitol (M) were selected and combined with choline chloride (CC) and betaine (B). Melt processing techniques (thermocompression and extrusion of selected system) were chosen for polysaccharide processing as simple, convenient, and solventless methods. Although sugar polyols-based eutectic mixtures are known from the literature [26,27,28], their starch plasticizing ability has not been practically studied so far. One exception is Zdanowicz and Johansson’s work [23], where S-based DES were used, but films based on PS and its derivative were prepared via casting from aqueous system. Moreover, there is lack of studies related to PS plasticized with different, mixed systems based on sugar alcohols.

**Scheme 1:**
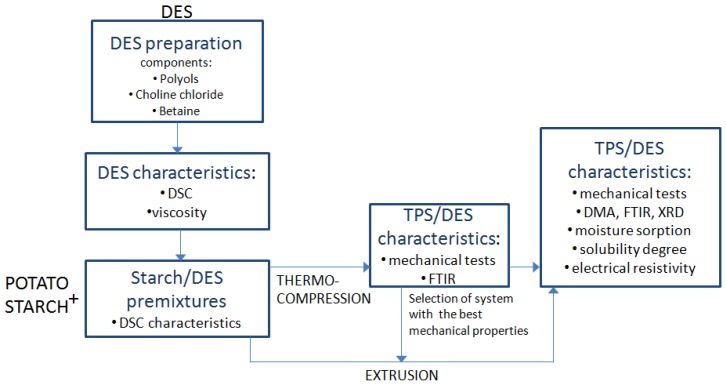
Flow chart of experimental design presented in this study.

Films were conditioned and characterized (visual, mechanical, FTIR). Influence of preconditioning parameters (storage time and temperature) on the properties of final products was also studied. Mechanical properties, morphology (XRD, DMA, FTIR), electrical resistivity and moisture content/sorption of the films obtained via two processing methods were investigated and compared. Moreover, for extrudates, the impact of preconditioning and different processing parameters, such as rotational speed, on final films properties was investigated. Flow chart of this study is presented in Scheme 1.

## 2. Materials and Methods

### 2.1. Materials

Native potato starch—PS (29.7% amylose content, 16.5 wt% moisture) was supplied by Zetpezet S.A. (Piła, Poland). Betaine—B (anhydrous, 99%), choline chloride—CC (≥98%, *T*_m_ 198 °C), maltitol—M (97%, *T*_m_ 145.5 °C), and xylitol—X (99%, *T*_m_ 92.5 °C) were purchased from Alfa Aesar (Kandel, Germany). Glycerol—G (pure, does not exhibited any transition at range from −90 °C in differential scanning calorimetry (DSC) curve) was purchased from Eurochem (Warsaw, Poland), sorbitol—S (99%, *T*_m_ 97.8 °C) from Acros Organics.

### 2.2. Preparation of DES

DES were prepared as follows: Selected components were placed in a glass reactor immersed in a water bath, heated up to 95 °C, and stirred until homogenous pellucid liquid was obtained; then the mixture was poured into a glass vial and placed in a vacuum chamber (105 °C, 250 milibar, 1 h) to remove moisture.

### 2.3. Preparation of PS/DES Premixtures and TPS/DES Thermocompressed Films

In the first step, starch/DES premixtures were prepared. DES and starch (35 wt parts of DES per 100 wt parts of dry starch) were placed in a mortar and ground to obtain a homogenous paste, then the composition was placed into PP vial, sealed, and stored for 24 h at ambient conditions. Some sealed samples were additionally treated, i.e., heated at higher temperature for certain time (standard heating procedure: 1 h at 80 °C—“ht” abbreviation with the sample name). Comparison films with pure polyols and CC were also prepared.

The premixtures were placed in steel frame between PET sheets and thermocompressed (hydraulic press ReMi-Plast s.c. PH10T, Poland) at 150 °C, 12 tons (153 bars) for 5 min, cooled down to 85 °C under pressure, and then stored in a climate chamber for 7 days before testing (25 °C, RH 50%). Table 1 presents description of abbreviations used for samples names.

### 2.4. Preparation of TPS/DES Extrudates

Preparation of TPS/DES extrudates was as follows: Premixture of starch and selected DES was processed (i) after 1 day storage (1d); (ii) 4 days storage (4d); (iii) as fresh (f) composition: 30 min after starch with DES mixing; and (iv) preheating (ht) (heating at 80 °C for 1 h and stored for 24 h before extrusion). The systems were extruded with laboratory twin-screw co-rotational extruder with *L*/*D* ratio 40:1, screw diameter 16 mm (PRISM Eurolab Digital). Temperature profile from the feed throat to the nozzle was 80/100/130/135/140/140/140/140/135/120 °C and screw speed was maintained at 50 or 100 rpm. After processing, extrudates were granulated (granulator Prism Varicut) obtaining ca. 3 mm pellets. After 14 days of storage at ambient conditions in sealed PE bags, pellets were thermocompressed at 150 °C, 12 tons for 2 min, cooled down under pressure to ca. 85–90 °C and stored in a climate chamber (25 °C, RH 50%) for 48 h before testing.

### 2.5. DSC

Phase transitions, i.e., starch plasticization, as well as influence of pretreatment (additional heating) and storage time on transition temperature, were investigated using DSC technique (Q100 TA Instruments, New Castle, USA). DES and PS/DES premixtures (before thermocompression) after 24 h storage were analyzed in aluminum hermetic pans with nitrogen as the cooling agent with heating rate 5 °C/min. Standard runs at the temperature range from −90 to 250 °C for DES and from 20 to 200 °C for PS/DES premixtures were applied.

### 2.6. Viscosity Measurement

Viscosity of DES was measured with a Brookfield RV rheometer (Stoughton, USA) equipped with cone/plate at 25 °C and with 20 and 50 rpm. Average viscosity values are placed in Table 1.

### 2.7. Mechanical Tests

Mechanical tests for TPS/DES films were performed using Instron 5982 (load cell 1 kN). The films (thickness 0.50–0.65 mm) were cut into 10 mm wide, 100 mm long stripes. The initial grip separation was 50 mm and the cross head speed was 10 mm/min. At least eight replicated samples for each system were tested and the mechanical parameters: elongation at break (*ε*), tensile strength (TS), and Young’s modulus (YM) with standard deviations were calculated with Bluehill 3 software.

### 2.8. XRD

Crystallinity of TPS/DES films was analyzed by X-ray diffraction (X’pert Pro, PANalytical, Almelo, The Netherland, operated at the CuK(alfa) wavelength 1.54 Å). The XRD analysis of the samples processed via two investigated methods was repeated after 12 months of storage in ambient conditions. Starch-based materials were analyzed in a form of film or as a powder directly obtained from extrudate to examine if an additional thermocompression affects TPS crystalline structure.

### 2.9. DMA Measurement

Dynamic mechanical thermal analysis (DMA Q800 TA Instruments, New Castle, USA) was used to measure and compare transition temperatures and storage modulus curves of TPS/G and TPS/DES films obtained from premixture and from extrudate. The measurements were carried out in a dual cantilever mode at frequency of 1 Hz, heating rate 3 °C/min, and temperature range from −80 to 140 °C.

### 2.10. FTIR-ATR Analysis

FTIR analysis was performed using the Nexus (Thermo-Nicolet, Waltham, USA) technique equipped with ATR. For each sample, 32 scans were taken from 4000–400 cm^−1^.

### 2.11. Electrical Resistivity Measurements

Electrical surface and bulk resistivities of the TPS/S:CC 1:2 films obtained via thermocompression, as well as extrusion and thermocompression, were measured and compared using an electrometer 6517A with electrode set Keithley 8009 (Keithley Instruments, Inc., Cleveland, USA).

### 2.12. Moisture Content, Moisture Sorption, and Water Sorption Degrees

Moisture content in TPS films was measured using moisture analyzer (Radwag MAX 60/NH, Radom, Poland). For moisture sorption and swelling tests, TPS/G and TPS/DES composite samples 20 × 20 mm were prepared. Samples were placed in a vacuum dryer (250 milibar) at 65 °C/24 h. Mass of dried samples, as well as stored in a climate chamber and kept at 25 °C/50% and 75% RH (for moisture sorption evaluation) or immersed in distilled water for 24h (for swelling behavior investigation), were determined.

### 2.13. Statistical Analysis

Statistical analysis of the mechanical test data for extruded samples was subjected to a one way analysis of variance and the significant difference was determined by the significance difference test (t-Student’s test).

## 3. Results

### 3.1. DES Characteristics

DES analysis results (i.e., transitions and degradation temperatures together with molar ratios of individual components, as well as viscosity and organoleptic characterization of appearance) is presented in Table 2.

All mixtures exhibited glass transitions at low temperatures except for M:CC 1:4, where a melting peak with minimum at ca. 80 °C was observed. The lowest *T*_g_ value, ca. −69 °C, was found for X:G 1:2. The higher G content in polyol-based eutectic mixture the less viscous DES. Heating of DES causes rapid drop of viscosity, therefore, preheating of some DES before addition into starch is advised to better mutual mixing. When X and S-based DES are compared, mixtures with S exhibit higher viscosity. It can be caused by a higher number of OH groups onto longer polyol chains are able to form more H-bonds between molecules, in cases of the latter. Comparing onset degradation temperature values, the highest thermal stability exhibited S:CC 2:1 (ca. 268 °C), whereas the lowest was S:G 1:2 (156 °C). According to Francisco et al. [29], mixtures that exhibit *T*_g_ instead of *T*_m_ are called “low transition temperature mixtures”; however, most related literature called CC mixtures with polyols DES [16].

### 3.2. DSC Study of Starch/DES Premixtures

An influence of additional pretreatment of compositions (heating and storage time, temperature) on transition occurred in starch (+moisture)/DES systems was investigated using the DSC technique. Relevant temperatures and enthalpy values (∆*H*) are presented in Table 3. Generally, it can be noticed that preheating led to lower onset temperature of transition and *T*_min_ and ∆*H*. This phenomenon is related with facilitation of thermocompression efficiency (see point 3.3.1).

The aim of this preliminary study was to investigate changes in starch/DES compositions to further conform of TPS processing parameters. Additionally, the analysis helped to partially explain why extra pretreatment facilitates thermocompression molding.

**Figure 1:**
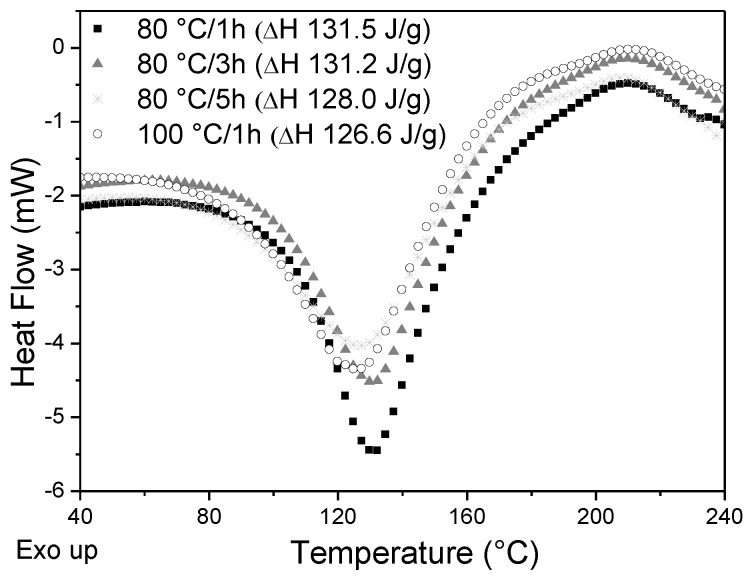
DSC curves for PS/S:CC 2:1 premixtures after different preheating parameters.

Figure 1 shows DSC curves for starch/S:CC 2:1 premixtures varied by preheating time and temperature.

It can be noticed that, with increasing heating time or temperature, ∆*H* slightly decreases (endothermic peaks become shallower). Moisture evaporation (see Supplementary Materials-SM, Figure S1), as well as swelling of starch granules, as the endothermic process absorbs heat, a consequence partially due to the swollen starch granules—with a lower degree of crystallinity, residual moisture, and polar DES presence after preheating—requires less heat to further transformation. Moreover, water molecules as starch moisture act as co-plasticizer and are bonded by DES, that is why do not evaporate so easily from the system. Thus, this additional treatment facilitates starch plasticization by thermocompression. As can be observed from the Figure 1, higher preheating temperature led to lower onset processing temperature (SM, Figure S2). Similar behavior can be observed for samples, where different storage time was studied. Figure S3 presents DSC curves for starch/S:G 1:2, where ∆H for composition after 4 days storage was almost twice lower (72.7 J/g) than after 1 day (140.5 J/g). For starch/polyols systems, the endothermic peak is related to *T*_m_ of sugar alcohols (SM, Figure S4), indicating that they require extra heat to melt and next to plasticize starch. Thus, systems with polyols need longer time or higher temperatures, or both, for effective polysaccharide modification.

### 3.3. Mechanical Tests

#### 3.3.1. Mechanical Results for Thermocompressed Films

Thermocompression tests were performed for starch not only with DES but also with individual polyols (G, X, S, and M), as well as with CC, for comparison their plasticizing ability (Table 3). The most common plasticizer, G, gave homogenous, transparent film with TS 4 MPa, *ε* 36.5%, and YM 135 MPa. X exhibited high tendency for crystallization in the starch matrix, whereas TPS with S or M alone were transparent but brittle. Heating of PS/S premixture before processing facilitated material plasticization, but *ε* of the film was still quite low (ca. 15%) with high YM (451 MPa), indicating an antiplasticizing effect. As our data show, CC alone is also not efficient plasticizer, even after prolonged conditioning time. When S:CC 2:1 was used as plasticizer (without preheating), TPS on its base exhibited almost the same high TS value (8.6 MPa) and insignificantly lower YM (428 MPa), but *ε* was twice higher than for TPS/S. Slightly lower TS near 7 MPa and high *ε* value above 69%, almost twice higher than for TPS/G, was observed for TPS/S:G 1:2. In other works related to starch processing with multicomponent plasticizers [13,30], mechanical properties parameters of TPS plasticized with S in G presence had values between these obtained for TPS/S and TPS/G. *ε* values of TPS materials with DES presented in this work are higher than for materials with individual components. Introduction of the mentioned polyols in eutectic liquid form, considered as “loose” plasticizers with greater total size, eases the polysaccharide chain’s mobility. Despite that plasticizer still contains molecules with a high amount of OH groups that interact with starch, there is no decrease of TS. In the literature, very often the mixed polyols were introduced in their solid form, so the mechanical properties values are between those for TPS with individual components. In Mikus et al.’s work [31], an increase of *ε* values for wheat TPS films plasticized with G and S mixture (12.5%/12.5%), in comparison with TPS plasticized with one type of polyol, was registered; however, TS (6.5 MPa) was much lower than for TPS/S (14.6 MPa).

**Figure 2:**
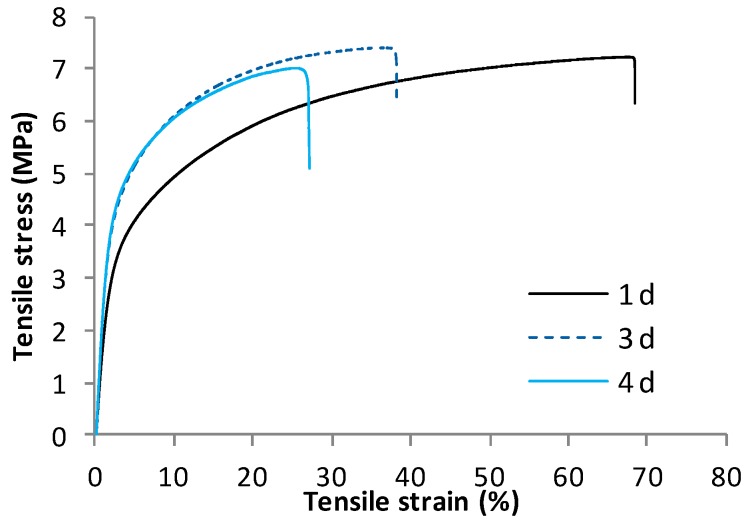
Mechanical properties for thermocompressed TPS/S:G 1:2 films with different storage time (at ambient temperature) before melt processing.

Preheating facilitates plasticization; however, it has an impact on final products properties. Influence of temperature and preheating time on TPS mechanical properties was investigated on examples of the selected DES: S:CC 2:1 and S:G 1:2 plasticizers (Figure 2, Figure S5). Increasing heating temperature highly influences *ε* values, and when it was increased from 80 to 100 °C, *ε* decreased (SM, Figure S5). The results show that 1 h of heating at 80 °C is sufficient to facilitate starch processing, and higher temperature or longer time of additional heating is not required. Partially swollen granules in DES presence after heating easily processed, affecting *ε* values. This behavior is supported by DSC results (Table 3, Figure 1, Figure S3). Interestingly, partial evaporation/migration of moisture acting as co-plasticizer from starch did not affect TPS elongation. Storage time of PS/S:G 1:2 premixture did not affect TS, but *ε* values diminished with a longer storage period, e.g., after 3 days of storage almost twice and, after 4 days, about an additional ca. 25% as compared with the sample after 1 day of storage (Figure 2). Changes in *ε* parameter are caused by stronger interaction between starch, moisture, and plasticizer molecules, limiting polymer chains movements. It can be correlated with DSC results presented on Figure S3, where ∆H is about two-fold lower for TPS/DES stored for 4 days but with higher *T*_min_ than for analogue stored for 1 day.

#### 3.3.2. Mechanical Results for Films Prepared from Extrudates

**Figure 3:**
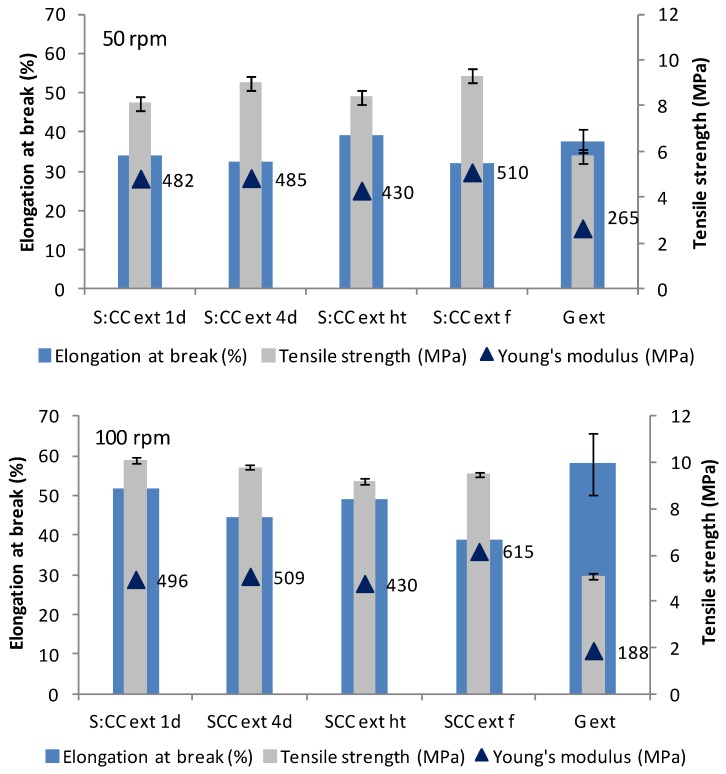
Mechanical properties of TPS/DES films plasticized with S:CC (2:1 molar ratio) prepared from extrudate (at two rotational speed of extrusion) and then thermocompressed.

Selected systems with the best mechanical properties, i.e., starch with S:CC 2:1 and starch with G for comparison, were processed via extrusion. Influence of the premixtures pretreatment and two rotational speeds of the processing on the final thermocompressed films properties was investigated. Mechanical tests results are presented in Figure 3.

Comparing samples obtained with two rotational speeds of extrusion, it can be found that the ones prepared with 100 rpm exhibited slightly higher TS and YM, as well as *ε*, values; however, the differences are not statistically significant (P > 0.05), except TS values for TPS/S:CC 1 d. It is noteworthy that YM values for TPS plasticized with S:CC are as a rule 2–3 times higher in comparison to the respective parameter values for films with G, and the differences are statistically significant (P < 0.05). Moreover, standard deviation values are, respectively, lower in the case TPS/S:CC samples extruded with 100 rpm than 50 rpm, suggesting better mixing during processing and homogeneity of the final material (SM, Table S1). TPS/G is an exception, where inverted relationship in TS values was observed. Despite the fact that *ε* value is slightly higher (58%), TS of TPS/G is two times lower than for TPS/S:CC 2:1. The highest TS (10.1 MPa) and *ε* (51.6%) were achieved for the TPS/S:CC 2:1 sample stored for 1 day before processing at 100 rpm. As data from Figure 3 show, processing of freshly prepared starch/DES composition is workable via extrusion, in comparison with thermocompression, where partially plasticized, brittle film was obtained (Table 4).

### 3.4. XRD of TPS/DES Films Pepared via Two Methods

**Figure 4:**
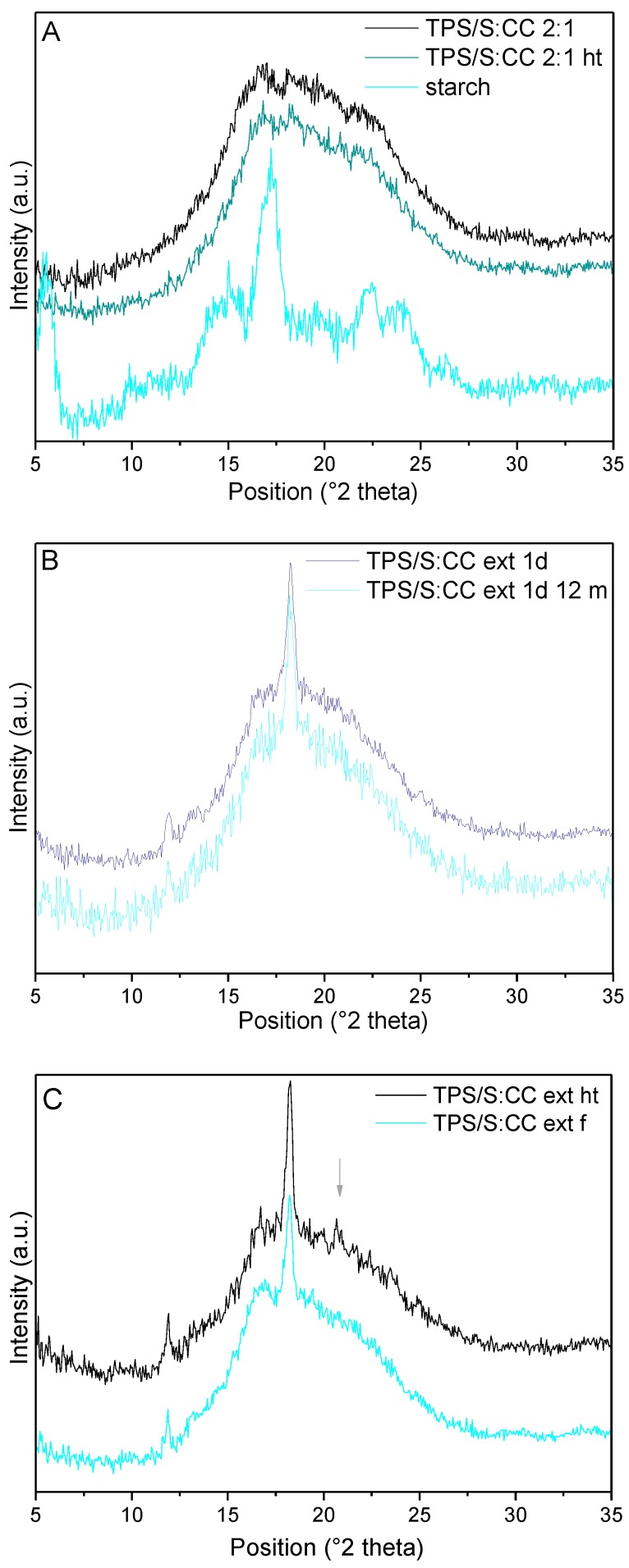
XRD patterns for (**A**) native PS (16.5 wt% moisture content) and thermocompressed starch/S:CC 2:1 including additional preheating (ht); (**B**) films from extruded starch/S:CC 2:1 after 14 days (1d) of storage and after 1 year (1d 12 m)—both from premixture processed after 1 day of storage; (**C**) films from materials with different pretreatment methods.

The influence of starch/S:CC 2:1 pretreatment, processing method, and storage time on starch crystallinity structure was investigated by XRD technique. Figure 4A presents diffractograms for native PS and TPS/S:CC 2:1 films (one after preheating, ht). Native PS exhibits characteristic peaks at 5.4, 17.1, 19.5, 22.5, 24.0, and 26.0°, forming B-type crystallinity. After thermocompression, these peaks got flattered, indicating more amorphous structure. There are no differences between the regular and preheated samples. Interestingly, XRD patterns for films prepared from extrudate are much different than only thermocompressed ones. Figure 4B presents diffractograms for extruded sample TPS/S:CC 1d measured 14 days after preparation and after one year. In comparison with only thermocompressed films, characteristic peaks of B-type structure disappeared, but a sharp peak with high intensity at 18.2° and much lower at 11.9° came from single helical V-type crystal structure, probably formed with amylose [32], are observed. From the literature, it is known that V-structures are formed from collapsed amylose helices forming complexes with adjuncts [33], e.g., G [34], lipid acids [35], or other compounds [36].

An intensity of peak at 18.2°, which is assigned to single helical structure and is characteristic for extruded TPS materials processed at higher temperatures with low water content [32], increased with the size of plasticizer molecule or total molecular DES structure (S:CC 2:1 > G:CC 2:1 > G) (SM, Figure S5B), suggesting increasing degree of the local complex formation [35]. This peak with space inside the helix can improve a polysaccharide chain’s mobility that is reflected in *ε* values, especially when we compare it with results for only thermocompressed TPS where the peak did not occur. Similar results were obtained in Li and Huneault’s work [37], where the intensity of V crystals increased with S content. Moreover, TPS/G and TPS/G:CC 2:1 have peaks at ca. 13.2° which are also assigned to V-type crystals [37,38], which are barely observed for TPS/S:CC 2:1. It can be related to higher hydrophilicity of TPS containing G. As it was found, TPS with G exhibited almost two-fold higher moisture content (8.2%) than in the case of that with S:CC 2:1 (4.5%), and the water molecules can be trapped between amylose helices.

There are no significant differences between XRD patterns for films prepared using various methods of the premixture treatment before extrusion. However, for preheated and freshly extruded samples, some visible peak at 17.1° and for TPS/S:CC ht barely visible at 20.7° can be observed (Figure 4C). During preheating, starch granules slightly swell (as DSC results show), and water molecules from starch moisture can migrate or being trapped in swollen polysaccharide helices, affecting some changes in complex structure reflected on XRD patterns.

There are no differences for the same sample analyzed after film preparation and after one year, which indicated lack of starch further recrystallization (SI, Figure S6B). The same results were observed for extruded samples obtained with other methods of pretreatment.

Comparing rotational speeds (50 and 100 rpm) for TPS/S:CC films, there were no significant differences on XRD pattern, similar to that between the powder and film form of TPS/S:CC samples (data not shown). It evidenced that subsequent thermocompression of the extrudate do not affect morphological structure (some V-type crystals still remained).

The last issue is the differences between crystallinity for the same compositions processed via two different methods. Shear forces during extrusion disrupt granules and pull out amylose, as it happens during starch gelatinization in water. Moreover, amylopectin is more sensitive to these forces and can degrade during processing in the extruder [39,40,41], unraveling amylose and, instead of double helices, the single helices with plasticizer are formed. Such a big difference in crystallinity of TPS systems in dependence on processing methodology was also reported in other work [42], where XRD of TPS composite films were obtained from aqueous solution or by thermocompression at 150 °C. In the XRD pattern for thermocompressed sample, sharp peaks at ca. 15, 18, and 23° are observed, whereas the diffractogram of TPS film obtained via the casting method has flattered shape. Contrary to plasticization with water only, where initial crystallinity disappears completely, presence of plasticizer, such as G, can stabilize rearranged structure in energetically unfavorable conformations of polysaccharides chains after extrusion [43].

### 3.5. DMA Measurements for TPS/DES Films

**Figure 5:**
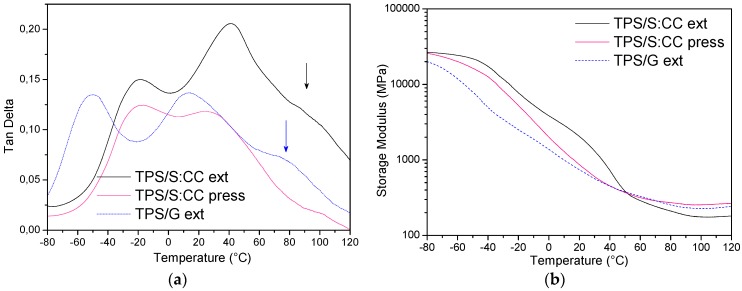
Tan delta and storage modulus for TPS/S:CC 2:1 films obtained by various processing methods (thermocompression-press and thermocompression after extrusion-ext) and for TPS/G after extrusion. (**a**) tan *δ* curves; (**b**) Storage modulus (E’)

TPS/DES films prepared via thermocompression only and extrusion followed by thermocompression were investigated by DMA. Additionally, TPS with G—conventional plasticizer—was taken to the comparison. In Figure 5a, tan *δ* curves are shown. The samples revealed two main relaxation regions of separated phases; the first with a maximum between −21 °C and −52 °C (β-relaxation, *T*_β_) related to secondary relaxation of the domain rich in plasticizer small molecules, and the second was above 14 °C (α-relaxation, *T*_α_) of starch-rich domain. Much higher temperatures of both relaxations exhibit films with S-based DES, rather than with G. This indicates a stronger interaction between starch with S:CC 2:1 than with G. Curves for TPS/DES films obtained by both processing methods (samples stored for 1 day before processing) are much different from each other. T_β_ are actually placed in the same area, but T_α_ of thermocompressed TPS/DES film (33.7 °C) is lower than for film from the extrudate (42.1 °C). Higher intensity of the α-relaxation peak evidences higher mobility of polymeric chains, caused by highly amorphous state of amylopectin and their partial mechanical degradation (see Section 3.4). It correlates with mechanical test results, and especially with *ε* values. Similar results, were reported in Mikus et al.’s [31] work, where mixed plasticizers (G and S) were used. There, a highly intense tan delta peak was found, but at lower temperatures, which can be related to lower TS values. Moreover, the discussed peak seems to be narrower, which indicated better miscibility of starch with plasticizer, in the result of extrusion [44]. At higher temperature range, some small and flat peaks (marked with arrows in Figure 5a) with peak onset temperature at ca. 80 and 58 °C for TPS/S:CC ext and for TPS/G ext, respectively, were observed. These peaks can be moisture molecule movements/evaporation (TPS/G with higher moisture content has a more noticeable peak than for S:CC containing material).

Storage modulus (*E*’) values (Figure 5b) at 25 °C for extruded TPS/S:CC, TPS/G, and thermocompressed TPS/SCC are 1661, 647, and 721, respectively, and are correlated to TS values.

### 3.6. FTIR-ATR Analysis

**Figure 6:**
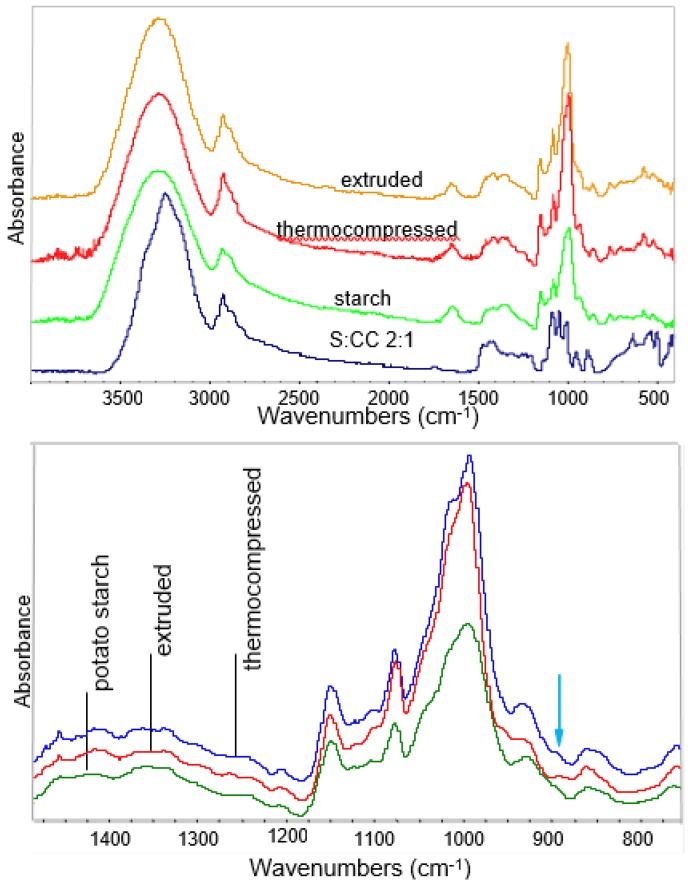
FTIR spectra for native PS, DES S:CC 2:1 and two TPS/S:CC 2:1 films obtained using different processing methods (thermocompression and extrusion followed by thermocompression).

FTIR spectra for native granular starch, DES alone S:CC 2:1, and two TPS/S:CC 2:1 films prepared via different processing methods are shown in Figure 6. Comparing starch and TPS/DES, there are some characteristic bands maxima shifts (SM, Table S2). A broad band with high intensity in the range 3600–3000 cm^−1^ is assigned to complex stretching free, inter-, and intramolecular hydroxyl groups [12]. For TPS/S:CC, there is a shift of peak maximum to lower wavenumber values (towards DES peak maximum). The shift is greater for extruded material than for those that are thermocompressed only (SM, Table S2). A band at range 3000–2800 cm^−1^ came from stretching C–H groups and, for TPS/DES, is a visible doublet evidencing DES presence. An extra subtle peak at 2853 cm^−1^ exhibited slightly higher absorbance for thermocompressed film than for that preliminary extruded. This one, and the greater shift of OH peak, can be related to better mixing of starch and plasticizer and their stronger interactions for extruded TPS/S:CC. Starch and TPS peaks in fingerprint range 1200–900 cm^−1^ related to stretching C–O and C–C, as well as bending C–O–H groups from anhydroglucose units, can be seen [45]. Here, a slight shift, but towards higher wavenumber values, was also observed (SM, Table S2), indicating interactions weakening of external OH starch chains and forming new ones with plasticizer. Moreover, intensity of this region is higher for both TPS films than for native granular starch. The higher intensity of short-range region, especially the 1022 cm^−1^ peak, was the more amorphous starch [46]. These results correspond with XRD data. Interestingly, films, especially for that based on preliminary extruded material, can be seen with uncovered peak at 891 cm^−1^. In the literature, there is a scarce discussion or explanation about its origin. According to [47], it can be assigned to ß-glucosidic linkages that are absent in starch or to C–Cl groups; however, in our work, this peak was also observed for TPS plasticized with other S-based DES (e.g., S:G 1:2), which do not contain chloride in their structure (data not shown).

S and their eutectic mixtures exhibited some peak at about 886 cm^−1^, so the most probable explanation is that this peak came from sugar alcohol but with some shift towards higher wavenumbers. It is characteristic that the peak is visible only for S (for example, in the case of DES mixtures with X, which also shows peak at 886 cm^−1^—there are no relevant peaks). A small peak in this wavenumber range has been observed in Vu and Lumdubwong’s work [48] for starch plasticized with S (film obtained via casting method) independently on polysaccharide origin, but it is not commented there.

### 3.7. Electrical Resistivity of TPS/DES Films

Results for surface and bulk electrical resistivity measurements for selected TPS films plasticized with S:CC 2:1 are presented in Table 5. Generally, there is no significant difference between the results. The highest values were observed for thermocompressed film that had been preheated before processing. All data indicating conductive properties (except for thermocompressed TPS/S:CC 2:1 ht, which exhibit dissipative activity) resulted from the presence of the ionic nature of CC. Additional thermal treatment of starch/DES premixture before thermocompression can enhance interactions between CC and hydroxyl groups of starch chains, leading to a decrease of Cl^−^ mobility; however, mixing with stress forces during extrusion and formation of amorphous structure resulted in disrupting these bonds.

### 3.8. Moisture Content, Moisture Sorption, and Water Sorption

All analyzed TPS films were kept in a climate chamber for 7 days before testing. Moisture content values in these samples are presented in Table 2. All TPS/DES films exhibit lower moisture content than TPS/G film, even if they contain hygroscopic CC. Comparing TPS/X:G 2:1 with TPS/S:G 2:1, the former had lower moisture content. It can be caused by a longer chain of polyol and a higher amount of OH groups strongly interacted with polysaccharide chains.

Similar results, i.e., an influence of molecular size of plasticizer on TPS sorption properties, were observed in other work [9]. Results of preliminary investigation of moisture content, moisture sorption, and water sorption degrees for TPS/S:CC and TPS/G obtained with two studied method are compared in Table 6. Moisture sorption is lower for films plasticized with DES S:CC 2:1 than for TPS/G. Comparing processing methods, extruded, and then thermocompressed films, exhibited a lower sorption degree than only thermocompressed at RH 50%; however, this difference is equalized at RH 75%. There is no significant difference in water sorption degree between films with two types of plasticizers; nevertheless, extruded samples exhibited a lower water sorption degree. This can be caused by better mixing of plasticizer in the starch matrix, as well as the presence of some local complexes formed with plasticizer molecules and helical regions of polysaccharide chains (see XRD results).

## 4. Conclusions

In the presented work, for the first time, sugar alcohol-based DES were used for PS plasticization via hot melting methods: thermocompression and extrusion with subsequent thermocompression. Influence of starch/DES premixtures conditioning (preheating temperature and storage time) before processing on final films properties was also investigated. Additional preconditioning of starch/DES mixtures facilitated starch plasticization and influenced mechanical properties, especially on elongation at the break of TPS/DES films—Samples modified with S-based DES exhibited high TS comparable to TPS with S only but with twice higher *ε* value. Films with S:CC 2:1 prepared from extrudate have better mechanical properties than those only thermocompressed. Maximum values of TS, *ε*, and YM for films based on extrudates attained ca. 10 MPa, 52%, and 616 MPa, respectively. XRD results pointed out amorphous morphology, but V-complex of amylose with DES after extrusion is formed. Intensity of this peak was dependent on plasticizer total molecular/structure size. All applied techniques (mechanical tests, XRD, DMA, and FTIR), indicated that S-based DES forms stronger interactions with carbohydrate polymer chains than G, leading to better mechanical properties (higher TS, as well as *ε*) and inhibited recrystallization of B-type semicrystallinity of PS (studied up to one year).

Generally, using sugar alcohol-based DES allows the formation of TPS materials with better mechanical properties and lower hygroscopicity features than TPS plasticized only with polyols. Due to the non-toxic, cheap character of DES components, such materials can be applied in food packaging, or even as edible films.

## Figures and Tables

**Table 1:** Description of abbreviations used with samples names.

Abbreviation	Description of Sample (Starch/Plasticizer Premixture) Conditioning
1 d	Storage for 24 h in sealed vial at ambient conditions before processing
4 d	Storage for 4 days in sealed vial at ambient conditions before processing
f	Processing after (about 30 min) premixture preparation
ht	Heating at 80 °C/1h in sealed vial and storage for 1 day before processing
ext	Extruded sample

**Table 2:** Transition temperatures of deep eutectic solvents (DES) analyzed by differential scanning calorimetry (DSC) and optical characterization.

DES Acronyms with Components Molar Ratios	Transition Temperature (°C)	Onset Degradation Temperature (°C)	Viscosity at 25 °C/80 °C (mPas)	DES Appearance at Room Temperature
X:CC 2:1	*T*_g_ −54.1	234.0	2420/82	Colorless, tendency to crystallization
X:G 1:2	*T*_g_ −69.1	216.0	1070/160	Colorless
S:CC 2:1	*T*_g_ −29.1	267.6	11,500/320	Slightly yellowish
S:CC 1:2	*T*_g_ −53.4	235.0	n.d./130	Colorless, tendency to crystallization
S:G 1:1	*T*_g_ −36.2	160.4	a.d.l./352	Slightly yellowish
S:G 1:2	*T*_g_ −53.4	156.0	1560/82	Slightly yellowish
S:B 2:1	*T*_g_ −19.1	191.1	n.d.	Yellowish, glassy state
M:CC 1:4	*T*_m_ 80.6	242.1	n.d.	Colorless, tendency to crystallization
M:G 1:6	*T*_g_ −65.1	227.0	850/71	Colorless, tendency to crystallization

*T*_g_—glass temperature; *T*_m_—melting temperature; n.d.—not determined; a.d.l.—above detection limit; X—xylitol; CC—choline chloride; G—glycerol; S—sorbitol; B—betaine; M—maltitol.

**Table 3:** DSC characteristics for potato starch (PS)/DES premixtures after 24 h of storage.

Sample	PS/DES Premixtures (Pastes Investigated before Thermocompression)		
	*T*_o_ (°C)	*T*_min_ (°C)	∆*H* (J/g)
PS/G	63.2	104.0	91.1
PS/X	88.4 ^a^	93.4 ^a^	147.5 ^a^
PS/X ht	50.9	98.0	223.0
PS/X:CC 2:1	68.1	100.6	114.5
PS/X:G 1:2	121.4	136.3	158.1
PS/S	81.5 ^a^	94.0 ^a^	163.0 ^a^
PS/S ht	38.5	83.2	153.6
PS/S:CC 2:1	86.6	117.0	207.5
PS/S:CC 2:1 ht	76.5	128.9	131.2
PS/S:CC 1:2	107.4	130.4	139.4
PS/S:B 2:1	81.9	118.7	320.8
PS/S:B 2:1 ht	37.3	76.1	156.1
PS/S:G 1:1	114.8	126.6	210.7
PS/S:G 1:1 ht	60.8	116.2	68.4
PS/S:G 2:1	112.7	134.5	140.5
PS/M	41.9	87.3 ^b^	291.3
PS/M ht	40.4	79.1 ^b^	186.6
PS/M:CC 1:4 ht	96.4	122.3	36.8
PS/M:G 1:6	44.2	86.3	95.2
PS/CC 2d	91.7	129.4	285.9
PS/CC 4d	100.1	129.7	291.3

*T*_o_—onset temperature of endothermal transition; *T*_min_—minimum temperature of endothermic peak; ∆*H*—enthalpy of endothermic peak; ht—heated sample (80 °C/1 h). ^a^*T*_m_ of polyol was observed; ^b^ sharp endothermic peak at ca. 151 °C assigned to M melting; *T*_min_ and ∆*H* for starch are 89.8 °C and 322.7 g, respectively.

**Table 4:** Mechanical properties of thermoplasticized compositions (standard deviation values in the brackets).

Sample	Results for Thermoplastic Starch (TPS)/DES Films Obtained via Thermocompression				
	Moisture Content (%)	Tensile Strength (TS) (MPa)	Elongation at Break (%)	Young’s Modulus (MPa)	Remarks
TPS/G	10.7 (0.4)	4.0 (0.10)	36.5 (4.3)	135 (11.3)	Transparent, flexible
TPS/X	n.m	Increasing crystallization during storage			
TPS/X ht	n.m.				
TPS/X:CC 2:1	n.m.	Transparent but brittle			
TPS/X:G 1:2	7.6 (0.1)	6.2 (0.24)	63.5 (10.2)	217 (15.1)	Transparent, flexible ^a^
TPS/S	n.m.	Brittle/partially plasticized			
TPS/S ht	5.0 (0.4)	8.7 (0.42)	15.1 (2.8)	451 (83.1)	Unplasticized edges
TPS/S:CC 2:1	6.1 (0.2)	8.6 (0.44)	33.4 (2.0)	428 (47.6)	Unplasticized edges
TPS/S:CC 2:1 ht	5.9 (0.2)	7.9 (0.54)	37.1 (2.7)	334 (40.9)	Unplasticized edges
TPS/S:CC 1:2	n.m.	Brittle			
TPS/S:B 2:1	n.m.	Brittle			
TPS/S:B 2:1 ht	5.2 (0.4)	8.1 (0.42)	18.8 (3.7)	537 (42.4)	Unplasticized edges
TPS/S:G 1:1	n.m.	Partially plasticized, brittle			
TPS/S:G 1:1 ht	6.2 (1.4)	7.7 (0.62)	38.1 (10.6)	361 (23.5)	Unplasticized edges
TPS/S:G 2:1	5.7 (0.9)	6.8 (0.10)	69.4 (7.6)	271 (15.1)	Transparent, flexible
TPS/M	n.m	Transparent but brittle			
TPS/M ht	n.m	Highly transparent but brittle			
TPS/M:CC 1:4 ht	9.9 (0.8)	7.8 (0.16)	37.3 (6.2)	354 (24.0)	Transparent
TPS/M:G 1:6	7.7 (0.7)	4.9 (0.30)	40.6 (7.1)	206 (26.0)	Transparent
TPS/CC 2d	n.m.	Partially plasticized, brittle core			
TPS/CC 4d	n.m.	2.9 (0.35)	8.5 (2.6)	205 (27.7)	Weak and matt

^a^ crystallization of plasticizer after few days storage.

**Table 5:** Surface and bulk resistivity of TPS/S:CC 2:1 films.

Sample	Surface Resistivity (Ω·m)	Bulk Resistivity (Ω)
Thermocompressed		
S:CC	8.6 × 10^6^	4.5 × 10^4^
S:CC ht	6.0 × 10^8^	1.0 × 10^6^
Extruded (100 rpm)		
S:CC f	9.9 × 10^6^	9.1 × 10^4^
S:CC 1d	7.6 × 10^6^	5.0 × 10^4^
S:CC ht	6.9 × 10^6^	4.0 × 10^4^

**Table 6:** Moisture sorption and swelling in distilled water of TPS/G and TPS/S:CC 2:1 films.

Sample	Moisture Sorption RH _50%_ (%)	Moisture Sorption RH _75%_ (%)	Water Sorption (%)
TPS/G press	10.3 (0.10)	30.0 (0.44)	104.8 (17.7)
TPS/S:CC press	6.2 (0.41)	24.0 (0.62)	108.5^d^ (7.3)
TPS/G ext	8.5 (0.21)	30.5 (0.86)	78.5 (1.5)
TPS/S:CC ext	5.9 (0.10)	22.9 (0.36)	83.2 (1.8)

d—partially defragmented.

## Supplementary Materials

The following are available online at https://www.mdpi.com/2073-4360/11/9/1385/s1, Figure S1. TG (solid) and DTG (dash) curves for PS/S:CC 1:2 premixtures; Figure S2. DSC curves for PS/S:CC 2:1 premixtures after different preheating parameters; Figure S3. DSC curves for PS/S:G 1:2 premixtures after different storage time; Figure S4. DSC curves for PS/polyol premixtures; Figure S5. Mechanical properties for thermocompressed films: A-TPS/S:CC 1:1 and B-TPS/S:CC 2:1with different preheating parameters; Table S1. Mechanical tests results for films based on preliminary extruded TPS/S:CC (2:1 molar ratio) films; standard deviations values in brackets; Figure S6. XRD patterns for TPS/S:CC films obtained after extrusion (100 rpm) with A: TPS/S:CC f (extruded composition without conditioning) after preparation and 12 months of storage; B: TPS obtained after extrusion with different plasticizers; Table S2. Bands maxima shifts in FTIR spectra of DES, native starch and TPS/S:CC 2:1 films obtained via two different methods.
