# Supplementary material for: Sugar Alcohol-Based Deep Eutectic Solvents as Potato Starch Plasticizers
Source: Polymers (Basel). 2019 Aug 23;11(9):1385. doi: 10.3390/polym11091385 (PMC6780061; doi:10.3390/polym11091385)
Supplement: Supplementary file 1 [file polymers-11-01385-s001.pdf]

SUPPLEMENTARY MATERIALS

Article

Sugar Alcohol-Based Deep Eutectic Solvents as Potato Starch Plasticizers

Magdalena Zdanowicz [1,*], Piotr Staciwa [1], Roman Jędrzejewski [2] and Tadeusz Spychaj [1]

1. West Pomeranian University of Technology, Szczecin, Faculty of Chemical Technology and Engineering, Polymer Institute, Ul. Pulaskiego 10, 70-322 Szczecin, Poland
2. West Pomeranian University of Technology, Szczecin, Faculty of Mechanical Engineering and Mechatronics, Institute of Materials Engineering, Al. Piastow 10, 70-310 Szczecin, Poland

* Correspondence: Magdalena.Zdanowicz@zut.edu.pl

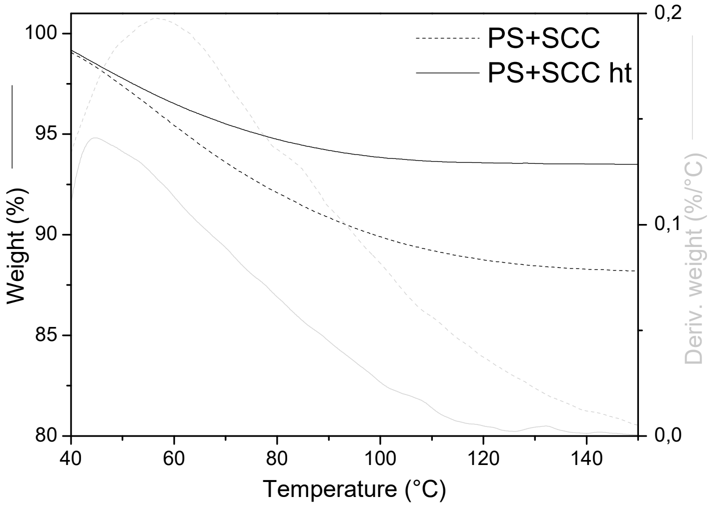

Figure S1. TG (solid) and DTG (dash) curves for PS/S:CC 1:2 premixtures. (Thermal stability of starch/DES premixtures was investigated using TGA: Q500, TA Instruments. Tests were performed on platinum pans under 25 mL/min air flow at a heating rate 10 °C/min).

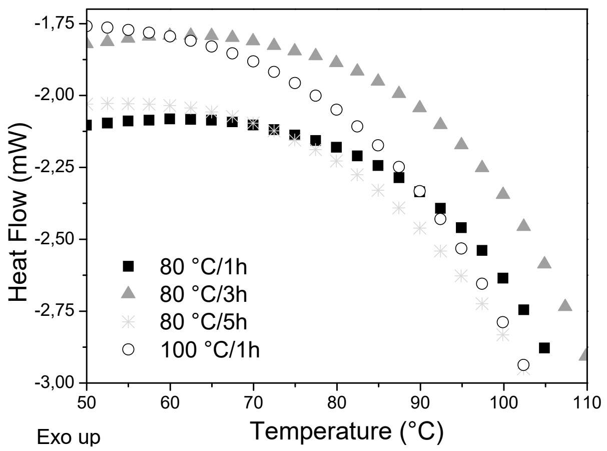

Figure S2. DSC curves for PS/S:CC 2:1 premixtures after different preheating parameters.

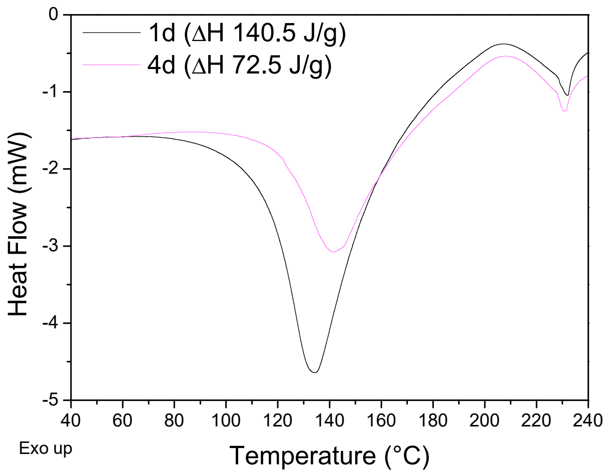

Figure S3. DSC curves for PS/S:G 1:2 premixtures after different storage time.

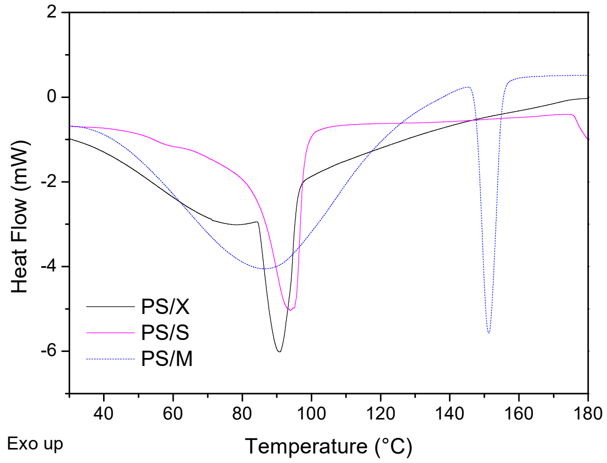

Figure S4. DSC curves for PS/polyol premixtures.

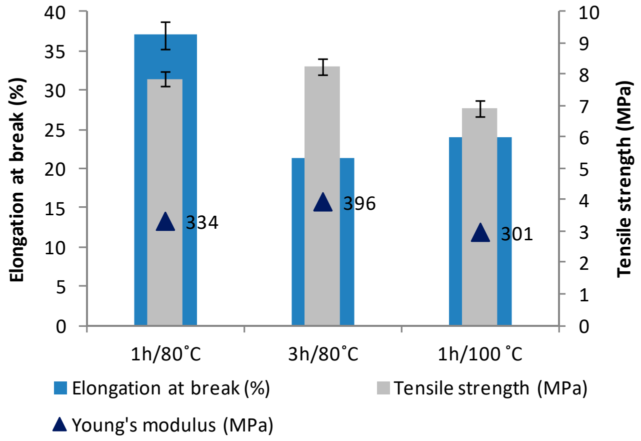

Figure S5. Mechanical properties for TPS/S:CC 2:1 thermocompressed films with different preheating parameters.

Table S1. Mechanical tests results for films based on preliminary extruded TPS/S:CC (2:1 molar ratio) films; standard deviations values in brackets.

Sample	Tensile strength (MPa)	Elongation at break (%)	Young's modulus (MPa)
		**50 rpm**	
TPS/S:CC 1d	8.1 (0.70)	34.1 (17.3)	482 (37.0)
TPS/S:CC 4d	9.0 (1.60)	32.3 (15.6)	485 (14.6)
TPS/S:CC ht	8.5 (1.40)	39.3 (12.3)	430 (58.2)
TPS/S:CC f	9.4 (0.83)	32.0 (14.0)	510 (25.3)
TPS/G 1d	5.1 (0.30)	37.7 (3.0)	266 (31.1)
		**100 rpm**	
TPS/S:CC 1d	10.1 (0.30)	51.6 (8.2)	496 (12.7)
TPS/S:CC 4d	9.8 (0.39)	44.6 (11.8)	509 (46.2)
TPS/S:CC ht	9.2 (0.34)	48.9 (5.5)	478 (76.4)
TPS/S:CC f	9.5 (0.29)	39.0 (9.4)	616 (30.0)
TPS/G 1d	5.8 (0.30)	58.0 (7.8)	188 (11.2)

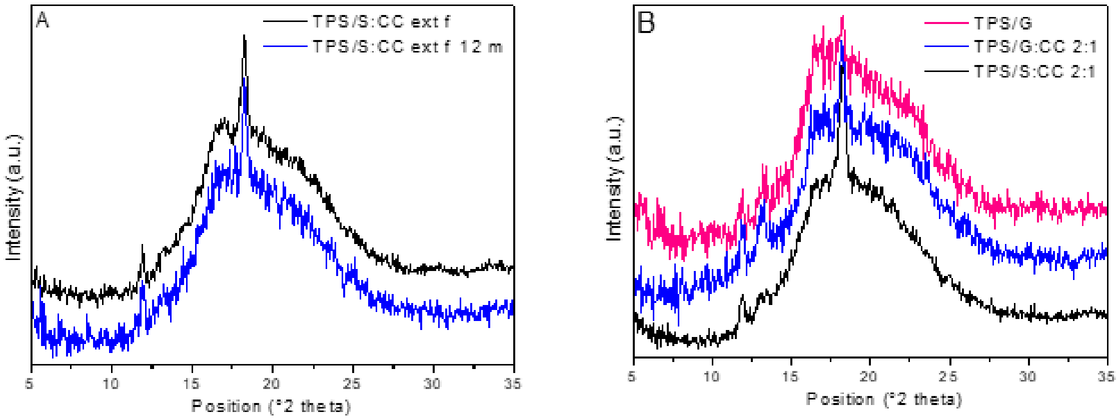

Figure S6. XRD patterns for TPS/S:CC films obtained after extrusion (100 rpm) with A: TPS/S:CC f (extruded composition without conditioning) after preparation and 12 months of storage; B: TPS obtained after extrusion with different plasticizers.

Table S2. Bands maxima shifts in FTIR spectra of DES, native starch and TPS/S:CC 2:1 films obtained via two different methods.

**Band range (cm^{-1})**	**Assigned groups**	**DES**	**Starch**	**Thermocompressed**	**Extruded and thermocompressed**
3600–3000	OH streching	3252.3	3290.0	3284.4	3278.5
3000–2800	C–H streching	2869.0	-	2853.1	2853.1
1100–900	C–O streching	-	992.0	995.7	992.6
